# Lithium-Ion Charged Polymer Channels Flattening Lithium Metal Anode

**DOI:** 10.1007/s40820-023-01300-5

**Published:** 2024-01-08

**Authors:** Haofan Duan, Yu You, Gang Wang, Xiangze Ou, Jin Wen, Qiao Huang, Pengbo Lyu, Yaru Liang, Qingyu Li, Jianyu Huang, Yun-Xiao Wang, Hua-Kun Liu, Shi Xue Dou, Wei-Hong Lai

**Affiliations:** 1 Hunan Provincial Key Laboratory of Thin Film Materials and Devices, School of Material Sciences and Engineering, Xiangtan University, Xiangtan, 411105 People’s Republic of China; 2 Guangxi Key Laboratory of Low Carbon Energy Materials, School of Chemical and Pharmaceutical Science, Guangxi Normal University, Guilin, 541004 People’s Republic of China; 3 Institute for Superconducting and Electronic Materials, Australian Institute of Innovative Materials, University of Wollongong, Innovation Campus, Squires Way, North Wollongong, NSW 2500 Australia; 4 Institute of Energy Materials Science, University of Shanghai for Science and Technology, Shanghai, 200093 People’s Republic of China

**Keywords:** Polymer ionic channel, Li metal batteries, Artificial protective layer, Uniform Li deposition, Electrochemical performances

## Abstract

The concentration difference in the near-surface region of lithium metal is the main cause of lithium dendrite growth. Resolving this issue will be key to achieving high-performance lithium metal batteries (LMBs). Herein, we construct a lithium nitrate (LiNO_3_)-implanted electroactive β phase polyvinylidene fluoride-*co*-hexafluoropropylene (PVDF-HFP) crystalline polymorph layer (PHL). The electronegatively charged polymer chains attain lithium ions on the surface to form lithium-ion charged channels. These channels act as reservoirs to sustainably release Li ions to recompense the ionic flux of electrolytes, decreasing the growth of lithium dendrites. The stretched molecular channels can also accelerate the transport of Li ions. The combined effects enable a high Coulombic efficiency of 97.0% for 250 cycles in lithium (Li)||copper (Cu) cell and a stable symmetric plating/stripping behavior over 2000 h at 3 mA cm^−2^ with ultrahigh Li utilization of 50%. Furthermore, the full cell coupled with PHL-Cu@Li anode and LiFePO_4_ cathode exhibits long-term cycle stability with high-capacity retention of 95.9% after 900 cycles. Impressively, the full cell paired with LiNi_0.87_Co_0.1_Mn_0.03_O_2_ maintains a discharge capacity of 170.0 mAh g^−1^ with a capacity retention of 84.3% after 100 cycles even under harsh condition of ultralow N/P ratio of 0.83. This facile strategy will widen the potential application of LiNO_3_ in ester-based electrolyte for practical high-voltage LMBs.

**Supplementary Information:**

The online version contains supplementary material available at 10.1007/s40820-023-01300-5.

## Highlights

- The LiNO_3_-implanted electroactive β phase polyvinylidene fluoride-*co*-hexafluoropropylene was built as an artificial solid electrolyte interphase layer for dendrite suppression.
- The electronegatively charged polymer layer can capture Li ion on its surface to form Li-ion charged channels and recompense the ionic flux of electrolytes via continuous supply of Li ion.
- The modified Li anode achieved a long cycle life over 2000 h under ultrahigh Li utilization of 50% in symmetric cell and worked in full cell for 100 cycles at harsh condition of extremely low N/P of 0.83.

**Supplementary Information:**

The online version contains supplementary material available at 10.1007/s40820-023-01300-5.

## Graphical abstract

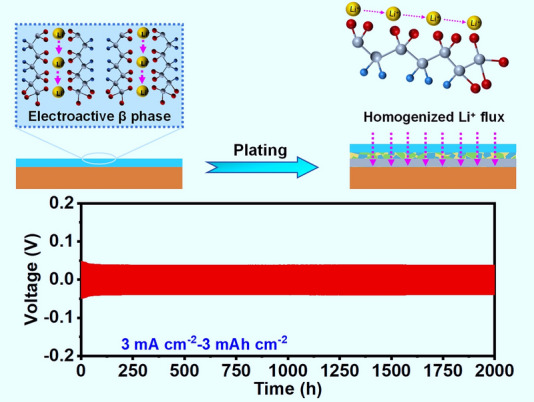

## Introduction

Li metal is an ideal anode material for next-generation rechargeable batteries because of its ultrahigh specific capacity (3860 mAh g^−1^) and lowest redox potential (− 3.04 V versus SHE) [1–4]. However, some fundamental challenges of Li metal in terms of low Coulombic efficiency (CE), short cycle lifespan, and safety concerns caused by the uncontrolled Li dendrite growth severely hinder its commercial application in lithium metal batteries (LMBs) [5–8]. Li metal with the features of high chemistry activity will easily react with organic electrolyte and Li slats to form heterogeneous solid electrolyte interphase (SEI) layer [9, 10]. The SEI layer plays a crucial role in battery safety, energy storage and cycle life, typically varying in width from several to hundreds of nanometers. Ideally, the SEI layer should have good ionic conductivity, perfect structural uniformity, and high elastic strength, which can promote Li^+^ transfer to the entire electrode surface quickly and uniformly, thus preventing Li dendrite growth and achieving uniform Li deposition [11, 12]. However, the native SEI layer with brittleness and instability severely impacts the Li plating/stripping processes [13–15]. During cycling, the rough and fragile SEI would crack under huge volume change, the fractured SEI will further expose the inner fresh Li and generate new fragile SEI [16]. The repeated break/generation of SEI will irreversibly consume Li metal and electrolyte, leads to low CE and short lifespan [17–19]. Moreover, the cracks will become “Hot Spot”, and Li^+^ near these active sites deposits rapidly, which accelerate the growth of dendrites [20]. Besides, the uncontrolled Li dendrite would pierce the separator and bring serious safety hazards [21, 22]. Therefore, it is necessary to build a stable SEI layer with high ionic conductivity and eminent elastic strength to suppress Li dendrite growth and expedite the commercialization process of LMBs.

In recent years, considerable efforts have been explored to construct robust SEI layer for Li dendrite inhibition through optimizing electrolyte configuration or building artificial SEI layer [23–27]. Previous studies reported that lithium nitrate (LiNO_3_) as electrolyte additive was a valid path to strengthen the interface stability for dendrite suppression [22, 23]. The LiNO_3_ has been proved to be critical electrolyte additive for Li–S batteries in restraining the “shuttle effect” of lithium polysulfides and improving interfacial stability of Li metal anode [29–32]. The NO_3_^−^ will degrade into Li^+^ conductors, Li_3_N and LiN_*x*_O_*y*_, which are beneficial for Li plating/stripping behavior [33, 34]. However, the application of LiNO_3_ additive for Li metal batteries is hampered by its extremely low solubility (< 0.05 M) in ester-based electrolyte [35, 36]. Nowadays, researchers have been devoted to enhancing the solubility of LiNO_3_ in ester-based electrolyte owing to the excellent film-forming capacity for dendrites suppression and potential application for high-voltage LMBs. On the one hand, considerable solubilizers, such as CuF_2_, pyridine, tin(II), *γ*-butyrolactone and tetraglyme, were developed to facilitate the dissolution of LiNO_3_ in carbonate-based electrolyte [28, 37–40]. However, the long-term cycle stability with high CE of LMBs was still hampered by the limited amount of LiNO_3_ dissolution (less than 0.3 M). On the other hand, using organic framework as a mediation may be a promising way to remarkably increase the utilization of LiNO_3_ in ester-based electrolyte [41–43]. For instance, Liu et al. introduced porous PVDF gel to encapsulate LiNO_3_ nanoparticles, which realized stable dissolution of LiNO_3_ during battery operation and exhibited high CE of ~ 98.1% over 200 cycles at a current density of 1 mA cm^−2^ under 1 mAh cm^−2^ [44]. Yang et al. prepared a poly(vinyl carbonate) organogel interlayer containing LiNO_3_ via electrospinning [39]. Thanks to the continuous release of LiNO_3_ from the interlayer, the cyclic stability of the Li metal anode was significantly improved to 300 cycles with high CE of 98.5%. Recently, a strategy for preparing ceramic-based composite protective layer enriched with LiNO_3_ was developed [45]. The slow release of LiNO_3_ into the electrolyte during cycling facilitated the sustained formation of stable SEI layer enriched Li_3_N that can effectively suppress Li dendrites. However, the development of LiNO_3_ for LMBs with high Li utilization in ester-based electrolyte remains challenging.

**Fig. 1:**
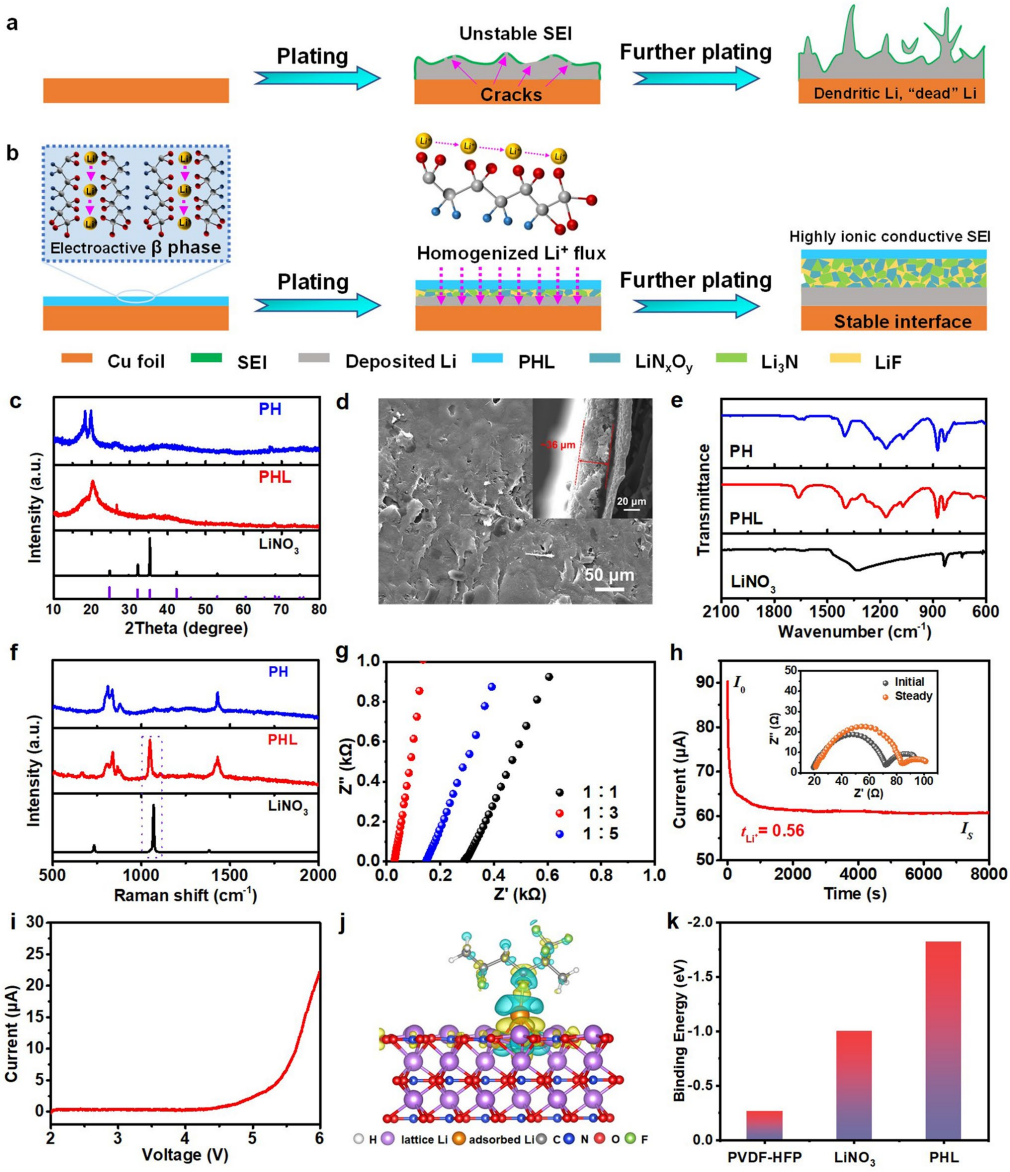
Schematic diagram of Li plating/stripping behavior for the **a** bare Cu and **b** PHL-Cu electrodes; **c** XRD pattern of LiNO_3_, PH and PHL; **d** SEM image of PHL-Cu electrode; **e** FT-IR and **f** Raman spectra of PH, PHL and LiNO_3_; **g** Chronoamperomtric curves and corresponding EIS before/after polarization of PHL-Cu@Li; **h** LSV profiles of PHL film with mass ratio of 3:1 in Li||SS cell (SS: stainless steel) at a scan rate of 0.2 mV s^−1^; **i** EIS plots of the SS|PHL|SS cells with various mass ratios at room temperature; **j** Differential charge density distribution of PHL; **k** Binding energy of Li atoms with PVDF-HFP, LiNO_3_, and PHL

In this study, a composite artificial SEI composed of PVDF-HFP (PH) and LiNO_3_ for Li metal anode is proposed. The PHL layer with high polarity can capture Li ions on its surface to form Li-ion charged channels, which serve as reservoirs to continually release Li ion during battery operation, thus lowering local Li-ion flux and achieving uniform Li-ion deposition (Fig. 1b). With the assist of the PHL layer, the Li||Cu cells exhibited high CE of 97.0% and 95.9% over 250 cycles at 0.5 and 1.0 mA cm^−2^, respectively. Under ultrahigh Li utilization of 50%, a long cycle life over 2000 h at a current density of 3 mA cm^−2^ could also be achieved in Li||Li symmetric cell. Besides, LMBs with LFP exhibit long-term cycle stability with high-capacity retention rate of 95.9% for 900 cycles and 84.3% with NCM under the harsh condition of extremely low N/P ratio of 0.83 for 100 cycles.

## Experimental

### Material

All the materials were obtained from commercial sources without any purification. Metallic Li foil was purchased from China Energy Lithium Co., Ltd., (Φ = 15.6 mm, 99.95%). Polyvinylidene fluoride-*co*-hexafluoropropylene (PVDF-HFP, M = 400,000) was obtained from Sigma. Tetrahydrofuran (THF, 99.9%), lithium nitrate (LiNO_3_, 99.99%), 1-methyl-2-pyrrolidinone (NMP, 99.5%), and dimethyl carbonate (DMC, 99%) were purchased from Macklin. Super-P, polyvinylidene fluoride, LiFePO_4_ (LFP) and LiNi_0.87_Co_0.1_Mn_0.03_O_2_ (NCM) cathodes were purchased from Guangdong Canrd New Energy Technology Co., Ltd. The ester-based electrolyte, 1 M LiPF_6_ in a mixture of ethylene carbonate (EC), ethyl methyl carbonate (EMC) and DMC (EC/EMC/DMC, 1:1:1 by volume) with 5 wt% fluoroethylene carbonate (FEC) as additive was supplied by Tinci Materials Technology Co, Ltd. All the operations involving Li metal anode and battery configuration were prepared inside an argon-filled Mikrouna glove box with both H_2_O and O_2_ concentrations below 0.1 ppm.

### Material Synthesis

The polymer-based composite film and polymer-based protective layer were prepared by a solution casting method. In brief, the PVDF-HFP and LiNO_3_ (PHL) with various mass ratio were dissolved in THF solution under magnetic stirring for 24 h. To prepared polymer-based composite film, the above solution was cast onto a Teflon plate and dried for 24 h to remove the THF solvent. For the PHL-Cu electrode, a certain amount of above solution was cast onto Cu foil after THF evaporation. The cathode electrode was obtained by mixing commercial cathode material (LFP or NCM), Super-P and polyvinylidene fluoride with a mass ratio of 8:1:1 using N-methyl pyrrolidinone (NMP) as a dispersant. The mass loading of LFP or NCM was around 3.6 and 6.0 mg cm^−2^, respectively. Specially, the mass loading of commercial NCM cathode was 18 mg cm^−2^.

### Material Characterization

The morphology and elemental distribution were observed by field emission scanning electron microscope (FE-SEM, Hitachi SU8010). Before operating, all the electrodes were rinsed with DMC to remove the residual Li salts and organic electrolyte after disassembling. The X-ray diffraction (XRD, SmartLab SE) technique was performed to detect the structure of the as-prepared samples in a 2θ range of 10°-80°. The compositional evolutions of the electrodes were monitored by X-ray photoelectron spectroscopy (XPS, Thermo ESCALAB 250XI). Fourier transform infrared (FT-IR) spectroscopy (Nicolet 6700) and Raman spectrometer (WiTech alpha300R) were conducted to analyze the microstructure of the as-prepared samples.

### Electrochemical Measurements

All the electrochemical performance tests of the as-prepared materials were evaluated using CR2016 or CR2032 type coin cells under Neware battery testing system (CT-4008T-5V50mA-164, Shenzhen, China).

The ionic conductivity (*σ*) of the PHL composite film was measured by electrochemical workstation (CHI660e, Shanghai Chenhua) in the frequency range of 10^5^–10^−1^ Hz at room temperature with stainless steel (SS)|PHL|SS cell. And the ionic conductivity was calculated according to the following Eq. 1:

1 $$\sigma = \frac{L}{{R_{{\text{b}}} S}}$$

where *L*, *R*_b_, and *S* represent the thickness of film, bulk ohmic resistance and area of electrode, respectively.

The lithium-ion transference number ($t_{{{\text{Li}}^{ + } }}$) was estimated by combination of chronoamperometry and EIS before/after polarization in symmetric cell, and then calculated with the following Eq. 2:

2 $$t_{{{\text{Li}}^{ + } }} = \frac{{I_{s} (\Delta V - I_{0} R_{0} )}}{{I_{0} (\Delta V - I_{s} R_{s} )}}$$

where Δ*V* is the potential difference applied during chronoamperometric step (10 mV), *I*_0_, *I*_s_ and *R*_0_, *R*_s_ are currents and interfacial resistances at the initial and the steady-state in the chronoamperometric step, respectively.

For the cyclic voltammetry (CV) measurement was performed on electrochemical workstation (Gamry, Reference 600+) using Li||Cu cells with the voltage range of − 0.3–0.6 or 0–2.5 V at a scan rate of 10 or 5 mV s^−1^. Electrochemical impedance spectroscopy (EIS) tests were conducted at an electrochemical workstation (CHI660e, Shanghai Chenhua) in the frequency range from 10^5^ to 10^−2^ Hz. Linear sweep voltammetry (LSV) tests were conducted on Li||SS cell at a scan rate of 0.2 mV s^−1^ in a voltage range of 2.0–6.0 V. Tafel test was measured on electrochemical workstation (Gamry, Reference 600 +) using Li||Li symmetric cell with a voltage range from − 0.2 to 0.2 V at a scan rate of 5 mV s^−1^.

The Li||Cu cells was applied to achieve CE testing, the Li metal was firstly plated onto the Cu substrate under various current density-capacity conditions and then stripped away up to 1 V. For the fabrication of the bare Cu@Li or PHL-Cu@Li anodes, 3 or 6 mAh cm^−2^ Li metal of was deposited within bare Cu or PHL-Cu electrode. The symmetric cells with bare Li or PHL-Cu@Li anode were performed on various current densities and deposition capacity to study the Li plating/stripping behavior. 50 μL of ester-based electrolyte was provided for the Li||Li and Li||Cu cells. For the full battery system, the bare Cu@Li or PHL-Cu@Li electrode was paired with LFP or NCM cathode. 50 μL of ester-based electrolyte was provided for the full cells. The optical cell with a transparent window was assembled by the bare Cu or PHL-Cu electrode and thin Li without using separator. The Li deposition process was recorded visually by optical microscopy at a current density 10 mA cm^−2^, and the play speed of the Movie was accelerated by almost 12 times.

### Calculation Method

All structure optimizations were performed at density functional theory (DFT) level using the projector augmented wave (PAW) method with electron exchange correlations described by the Perdew–Burke–Ernzerhof (PBE) functional within the generalized gradient approximation (GGA) scheme [46]. All calculations were performed employing Vienna Ab Initio Simulation Package (VASP) [47]. The energy cutoff of the plane-wave basis set has been consistently set to 500 eV. The convergence criteria of 0.05 eV Å^−1^ and 10^–4^ eV were used for the forces and energy during the geometry optimizations. The DFT-D3 correction was used to describe the dispersion contribution [48].

## Results and Discussions

### Synthesis and Characterizations of PHL Layer

The XRD analysis of PH, PHL and LiNO_3_ is compared in Fig. 1c. As displayed, the peaks of 18.25° and 19.88° were corresponded to the non-polar α and polar β phases (Fig. S1), respectively. As can be clearly seen, the intensity of non-polar α phase was reduced for the PHL, indicating that the introduced LiNO_3_ is beneficial for the formation of electroactive β phase, which may related to the ion–dipole interaction between ions and the polymer matrix [49]. The PHL composite films with mass ratios were conducted by XRD tests, as shown in Figs. 1c and S2. No peaks related to LiNO_3_ can be observed in the PHL films with mass ratios of 1:3 and 1:5. The absent peaks of LiNO_3_ indicate that the optimal LiNO_3_ can be successfully embedded into the polymer framework with dissociated state rather than crystalline state. However, with the LiNO_3_ content increased, the peaks related to LiNO_3_ (24.71°, 32.05°, 42.32°) was appeared (Fig. S2a). It can be seen form the SEM results (Fig. 1d), the PHL composite film showed a relative smooth surface with thickness of ~ 36 μm rather than porous structure (Fig. S3), verifying the complete merger of LiNO_3_ into the polymer matrix. Meanwhile, the optical photographs verified that the PHL films with optimal LiNO_3_ exhibit excellent flexibility and mechanical properties, as displayed in Fig. S4. As shown in the N_2_ adsorption/desorption isotherm, the surface area of PHL was 3 m^2^ g^−1^ (Fig. S5), further indicating that the LiNO_3_ was incorporated into the polymer matrix. The structural properties of PH, PHL and LiNO_3_ was analyzed by FT-IR (Fig. 1e) and Raman spectra (Fig. 1f). In the FT-IR spectrum, the characteristics vibrational peaks located at 837, 874 and 1275 cm^−1^ were ascribed to the polar β phase [50]. And the new peaks were appeared at 675 and 1664 cm^−1^ for the PHL film, which may be caused by the cross-linking with interaction between the Li^+^ and polar functional group of PH [51]. The pronounced Raman peaks at 800 and 837 cm^−1^ could be attributed to the Raman modes of non-polar α and polar β phases [52]. Apparently, the intensity of α phase reduced after introducing LiNO_3_. Compared to PH, a broader peak at 1435 cm^−1^ corresponding to β phase electroactive can be observed for the PHL, indicating lower crystallization. After merging into the polymer skeleton, the peak at 1070 cm^−1^ corresponding to LiNO_3_ undergoes slight shift to 1043 cm^−1^, which could be derived from the interaction between the −CF− functional group and Li ion.

Ionic conductivity is a crucial indicator for artificial SEI layer, which deeply affects rapid Li-ion transport and uniform Li deposition. As shown in Figs. 1g, S6 and Table S1, the ionic conductivity was conducted by EIS at room temperature via assembling SS|PHL|SS cells. And the calculated result revealed that the ionic conductivity of PHL composite film with rational mass ratio of 1:3 is 3.39 × 10^−4^ S cm^−1^. Therefore, the protective layer with high ionic conductivity is beneficial for uniform Li deposition at high current density. Chronoamperometry profile and EIS before/after polarization of the symmetric cell were depicted in Figs. 1h and S7. The Li^+^ transference number ($t_{{{\text{Li}}^{ + } }}$) of the PHL-Cu@Li is 0.56, which is prominently higher that of bare Li (0.27). Such high $t_{{{\text{Li}}^{ + } }}$, caused by the cross-linking with interaction between the Li^+^ and −CF− functional group (in accordance with the Raman and FT-IR results), is able to avoid anion depletion-induced strong electric fields and thus inhibit the growth of Li dendrites. Besides, the electrochemical stability of the PHL was monitored by linear sweep voltammetry (LSV) tests in Li||SS cell, as shown in Fig. 1i. The PHL films with mass ratio of 3:1 exhibited high anodic peaks (> 5.25 V), implying the reliability of PHL for LMBs during high-voltage operation. To examine the impact of the formation of the PHL on its affinity for Li ions compared to its constituent materials, we conducted DFT calculations. The corresponding differential charge density distributions upon the adsorption of Li over the PHL show a significant charge transfer from Li to PH and LiNO_3_ (Fig. 1j). These results further confirm the substantial interaction between Li and PHL. The lithophilicity of PHL, PH and LiNO_3_ was investigated based on the optimized geometries (Fig. S8), as shown in Fig. 1k. It is noteworthy that the binding energies of the atoms on different substrates increase sequentially: PVDF-HFP (− 0.26 eV) < LiNO_3_ (− 1.00 eV) < PHL (− 1.82 eV). Obviously, the PHL exhibits a higher affinity for Li compared to PH and LiNO_3_, indicating more Li diffusion channels, which is beneficial for the rapid Li-ion transport and achieve uniform Li deposition.

### Electrochemical Performances of PHL-Cu or PHL-Cu@Li Electrodes

**Fig. 2:**
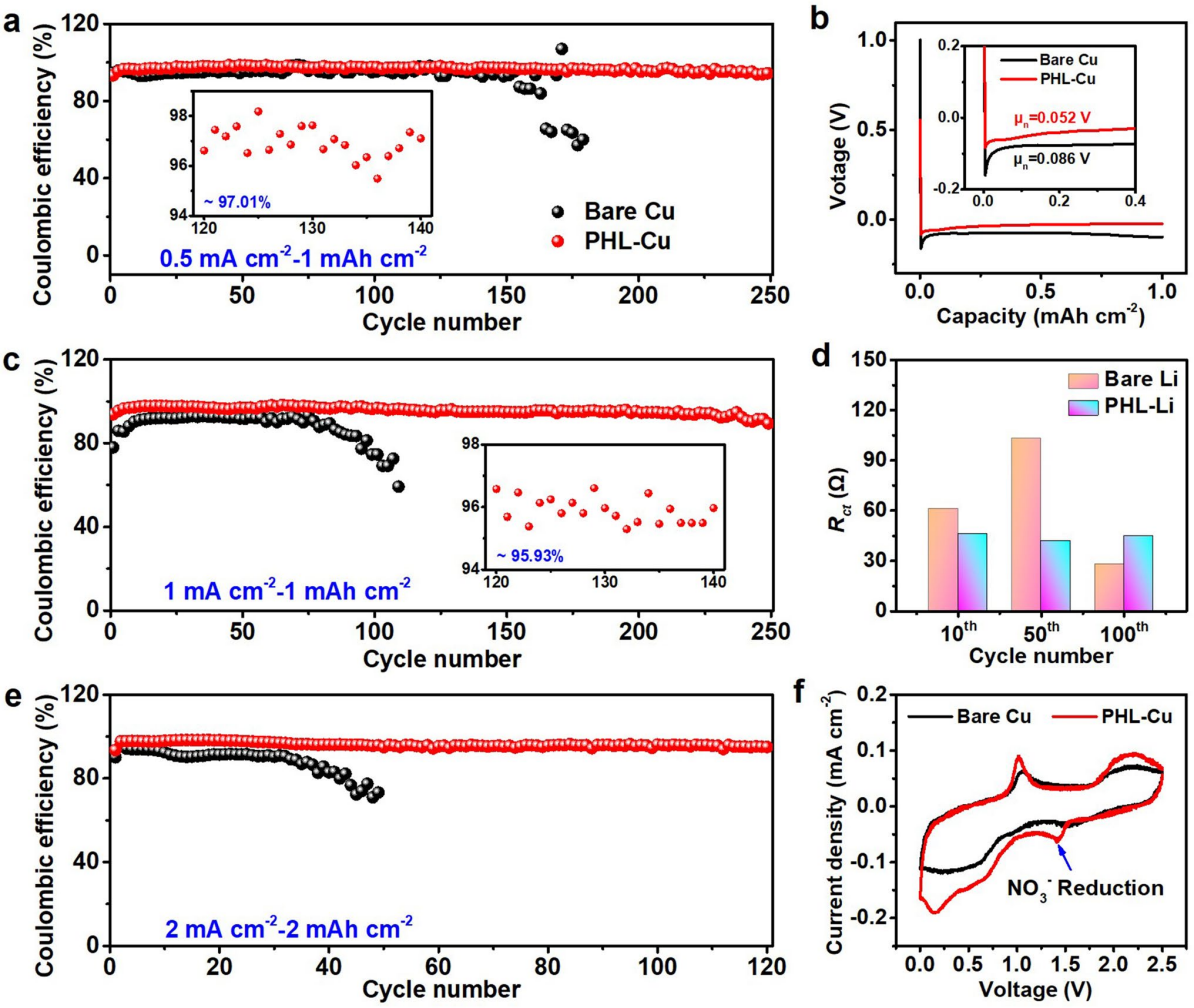
**a** CE and **b** initial plating profiles of Li||Cu cells with bare Cu or PHL-Cu electrode at 0.5 mA cm^−2^ under a areal capacity of 1 mAh cm^−2^; **c** CE of Li||Cu cells with bare Cu or PHL-Cu electrode at 1 mA cm^−2^ under a areal capacity of 1 mAh cm^−2^; **d** Charge transfer resistance (*R*_ct_) values of Li||Cu cells with bare Cu or PHL-Cu electrode after different cycles; **e** CE of Li||Cu cells with PHL-Cu electrode at 2 mA cm^−2^ under areal capacity of 2 mAh cm^−2^; **f** CV curves of Li||Cu cells with bare Cu or PHL-Cu electrode between a voltage window of 0 ~ 2.5 V at 5 mV s^−1^

CE is a vital parameter to appraisal the utilization efficiency of Li metal anode, which interprets as the ratio between the stripped Li and the plated Li during the plating/stripping process. The Li||Cu cells with bare Cu and PHL-Cu were assembled to monitor the CE under the various testing conditions. As shown in Figs. 2a and S9, a stable cycling of 250 cycles with a high CE of ~ 97.0% was achieved by PHL-Cu electrode in carbonate-based electrolyte, while the bare Cu electrode present a lower CE and worse lifespan at 0.5 mA cm^–2^ under a deposition capacity of 1 mAh cm^–2^. Figure 2b depicts the initial plating profiles of both bare Cu and PHL-Cu electrodes at 0.5 mA cm^–2^ to 1 mAh cm^–2^. The PHL-Cu showed a relatively low nucleation overpotential (*μ*_n_, calculated by the difference between the voltage dip and stable plateau) of 52 mV, which is significantly lower than that of the bare Cu electrode (86 mV), indicating lower nucleation energy barrier and faster kinetics nucleation process of Li on PHL-Cu substrate. When the current density increased to 1 mA cm^−2^, the cell with PHL-Cu contained outstanding stability for more than 250 cycles with average CE of 95.9% (Fig. 2c), which is superior to the bare Cu electrode (decay to 80% within 100 cycles). To demonstrate the superiority of the designed PHL layer, 0.257 M of LiNO_3_ (matched with the PHL layer) was introduced into the ester-based electrolyte as additive, as expected, the LiNO_3_ shows poor solubility in the ester-based electrolyte (Fig. S10), and the Li||Cu cell using LiNO_3_ as electrolyte additive presents low CE and short cycle lifespan (Fig. S11). In comparison to the bare Cu electrode, the PHL-Cu electrode exhibited lower interfacial impedance after different cycles (Figs. 2d and S12), implying a preferable electrochemical kinetics for Li plating/stripping behavior and in accordance with the Cyclic voltammetry (CV) results (Fig. S13). Even at the current density of 2 mA cm^–2^ under higher areal capacity of 2 mA cm^–2^, the cell with PHL-Cu electrode still sustained a stably cycling with high CE of ~ 95.4% for 120 cycles (Figs. 2e and S14), while the CE of cells with bare Cu appeared fading after only 30 cycles. The results clearly demonstrate that the PHL layer can markedly reduce the nucleation barrier of Li and enhance Li utilization efficiency during long-term cycling. CV was conducted to survey the formation mechanism of the SEI layer in Li||Cu cell at a scanning rate of 0.1 mV s^–1^ in a voltage range of 0–2.5 V. As depicted in Fig. 2f, the cell with PHL-Cu@Li anode displayed a broad peak at ~ 1.42 V versus Li/Li^+^, which could be ascribed to the reduction of LiNO_3_. The decomposition products, mainly nitrogen-containing species, are favorable for faster Li-ion transport and uniform Li deposition.

**Fig. 3:**
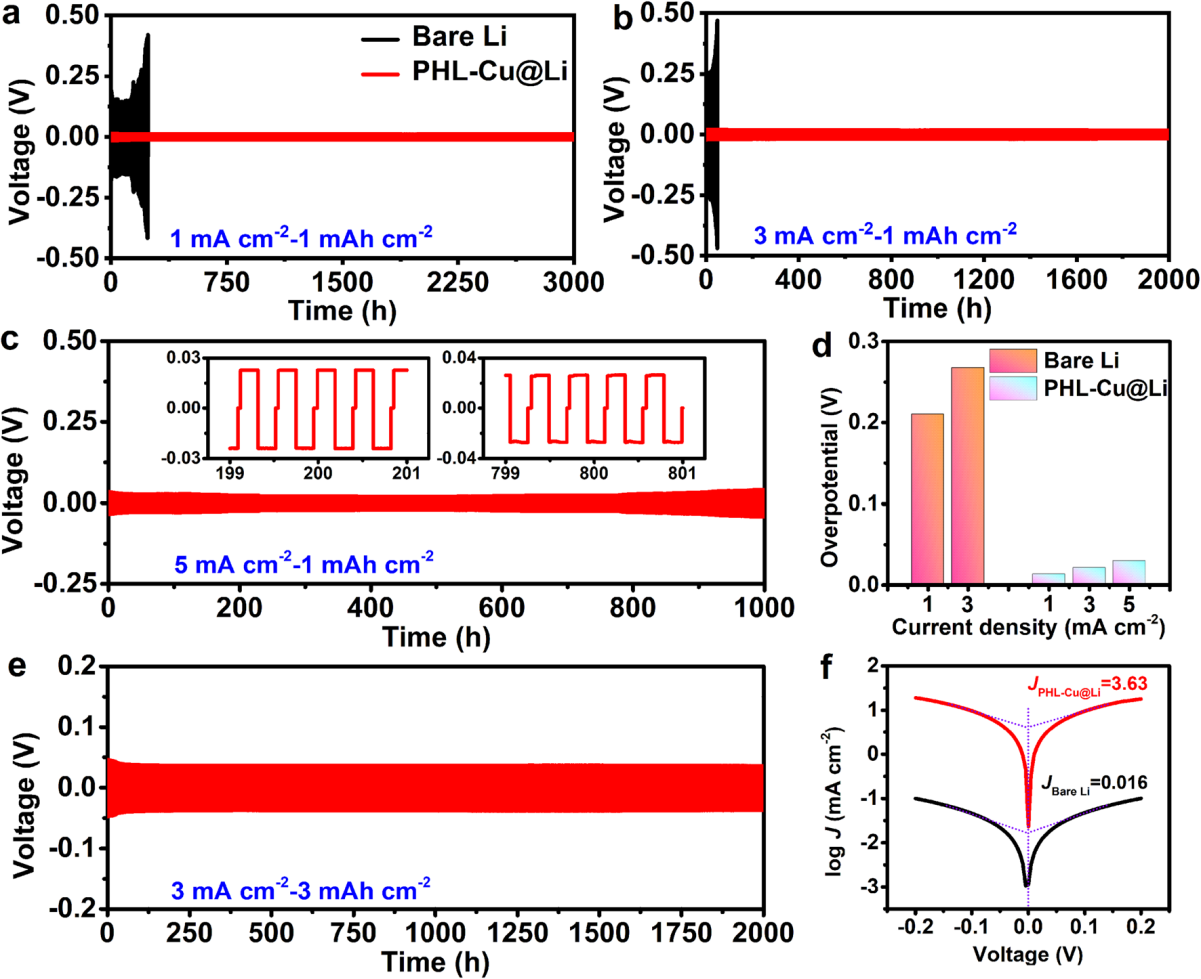
Voltage profiles of Li||Li symmetric cells using bare Li or PHL-Cu@Li at a fixed areal capacity of 1 mAh cm^−2^ under various current densities: **a** 1 mA cm^−2^, **b** 3 mA cm^−2^ and **c** 5 mA cm^−2^; **d** Overpotential of Li||Li symmetric cells using bare Li or PHL-Cu@Li; **e** Voltage profiles of Li||Li symmetric cells with PHL-Cu@Li at a current density of 3 mA cm^−2^ under high capacity of 3 mAh cm^−2^; **f** Tafel curves of Li||Li symmetric cells using bare Li or PHL-Cu@Li in the voltage range of − 0.2  to 0.2 V

To further evaluate the superiority of PHL layer under long-term cycling, the galvanostatic test of symmetric cells at various capacity-current density conditions was performed. First, 6 mAh cm^−2^ of Li was plated on the PHL-Cu electrode at 0.5 mA cm^−2^ to form the PHL-Cu@Li anode. Figure 3a, b depicts the Li plating/stripping profiles of Li||Li symmetric cells at current densities of 1 and 3 mA cm^−2^ under a fixed capacity of 1 mAh cm^−2^ in ester-based electrolyte. The bare Li anode delivered gradual increased overpotential with cycle number and failed after only 255 and 50 h, respectively, implying the information of fluctuant electrode/electrolyte interface due to the Li dendrites and “dead Li”. In contrast, the cells with PHL-Cu@Li anode displayed durable cycle lifespan (3000 h for 1 mA cm^−2^, 2000 h for 3 mA cm^−2^ and 1000 h for 5 mA cm^−2^) with low overpotential. Such extremely different results indicate a stable and flat SEI layer was built, which can be also demonstrated EIS tests (Fig. S15) after cycling. The excellent Li plating/stripping behavior with low overpotential (Fig. 3d) could be attributed to the stable electrode/electrolyte interface. The LiNO_3_, prestored in polymer framework, would continuously decompose into nitrogen-containing species for heightening the stability of SEI layer during operation. Moreover, the electroactive β phase with plentiful functional groups can cross-link with Li^+^, which not only further improve the interfacial stability but also expedite the Li-ion transport. Under the shield of the functional layer, the PHL-Cu@Li anode exhibited an impressive cycle stability for 2000 h with high Li utilization of 50% at 3 mA cm^−2^ (Fig. 3e). When the capacity rises to 4 mAh cm^−2^ (corresponding to a high Li utilization of 66.7%, Fig. S16), a stable Li plating/stripping behavior over 1000 h with small hysteresis voltage is well maintained for the PHL-Cu@Li anode. Besides, the Tafel plots (Fig. 3f) indicated that the exchange current density of PHL-Cu@Li anode (3.63 mA cm^−2^) is much higher than that of the bare Li anode (0.016 mA cm^−2^), implying the faster charge transfer of PHL-Cu@Li anode, which are in accordance with the chronoamperomtric and EIS results. Compared with previous reports on LiNO_3_ dissolution in carbonate electrolyte for Li metal anodes (Table S2), the PHL-Cu@Li anodes in this work present conspicuous advances in terms of long cycle life, high current density and high areal deposition capacity.

### Li Deposition Morphologies and Characterization of PHL Layer

**Fig. 4:**
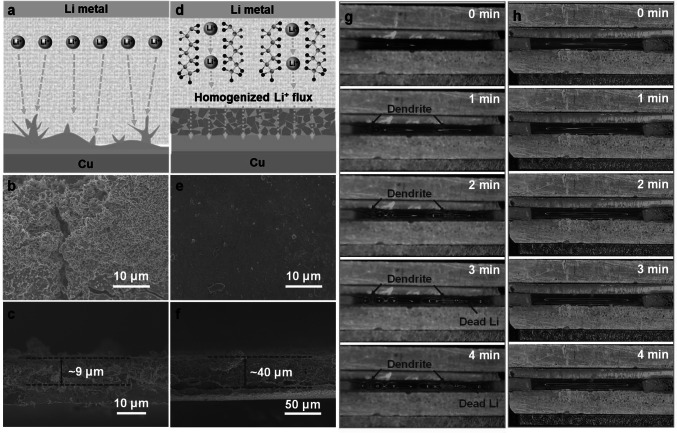
Schematic diagrams of Li plating behavior on **a** bare Cu and **d** PHL-Cu electrodes; SEM images of the **b,c** bare Cu and **e,f** PHL-Cu electrodes after 50 cycles at 1 mA cm^−2^; In situ optical microscopy images (captured from Movies S1 and S2) of the Li plating process on **g** bare Cu and **h** PHL-Cu electrodes at a high current density of 10 mA cm^−2^

To show the benefits of PHL layer, the Li deposition morphology evolutions on bare Cu and PHL-Cu electrodes were illustrated and investigated. For the bare Cu electrode (Figs. 4a and S17), the intrinsic protrusions and cracks of the bare Cu surface can cause Li-ion flux to gather in their regions and then induces the uneven deposition, leading to the accumulation of “dead Li” and rapid depletion of electrolyte during further cycling. With the aid of PHL layer (Figs. 1d, 4d), uniform and dendrite-free Li deposition can be realized, which is related to the following reasons: (1) The polymer matrix serves as a reservoir to release LiNO_3_ for gradually generation of nitrogen-containing species and then suppress Li dendrite growth; (2) The PHL layer with plentiful polar functional groups and high ion conductivity is capable of homogenizing Li-ion flux and facilitating uniform Li plating/stripping behavior. To further confirm the significance of the PHL layer, SEM was performed to visually observe the Li deposition morphology on bare Cu and PHL-Cu after 50 cycles at a current density of 1 mA cm^−2^ with a fixed capacity of 1 mAh cm^−2^. The bare Cu electrode exhibited a rough surface with plentiful Li dendrites, and the accumulated “dead Li” layer reaches 9 μm (Fig. 4b, c). In contrast, the PHL-Cu electrode maintains a flat and smooth surface without any dendrites, and the Li is uniform deposited into the PHL (Fig. 4e, f). From the EDS mapping of PHL-Cu electrode after 50 cycles (Fig. S18), the N and O elements were uniformly distributed on its surface, indicating the formation of stable interface layer. Besides, it's worth noting the PHL-Cu electrode still showed flat surface without any Li dendrites or “dead Li” after longer cycling (Fig. S19). The stark results demonstrate that the robust PHL layer can homogenize the Li-ion flux and accelerate interfacial ion transport, resulting in compact and dendrite-free Li deposition morphology.

In-situ optical microscopy was carried out to visually observe the dynamic morphology evolution of Li deposition. The Li||Cu cell with bare Li as anode and bare Cu or PHL-Cu as cathode was assembled without use of separator. The optical cells were conducted under a very high current density of 10 mA cm^−2^ to record the morphological evolution of deposited Li. Both of the bare Cu and PHL-Cu electrodes exhibited smooth and tidy surface in the initial phase (Fig. 4g, h, captured from Movies S1 and S2). For the bare Cu electrode, the dendrite starts to appear on the surface of Cu substrate just after 1 min of deposition. With the increase of deposition time, the “dead Li” and dendritic Li massively aggregate on bare Cu surface due to the lack of robust SEI layer. During early stage, the cell with PHL-Cu electrode showed uniform Li deposition with smooth morphology. And the flat surface of PHL-Cu without observable Li dendrite or “dead Li” could be maintained during the further deposition process, demonstrating that the PHL layer is useful to guide uniform Li deposition and reduce interfacial adverse reaction.

**Fig. 5:**
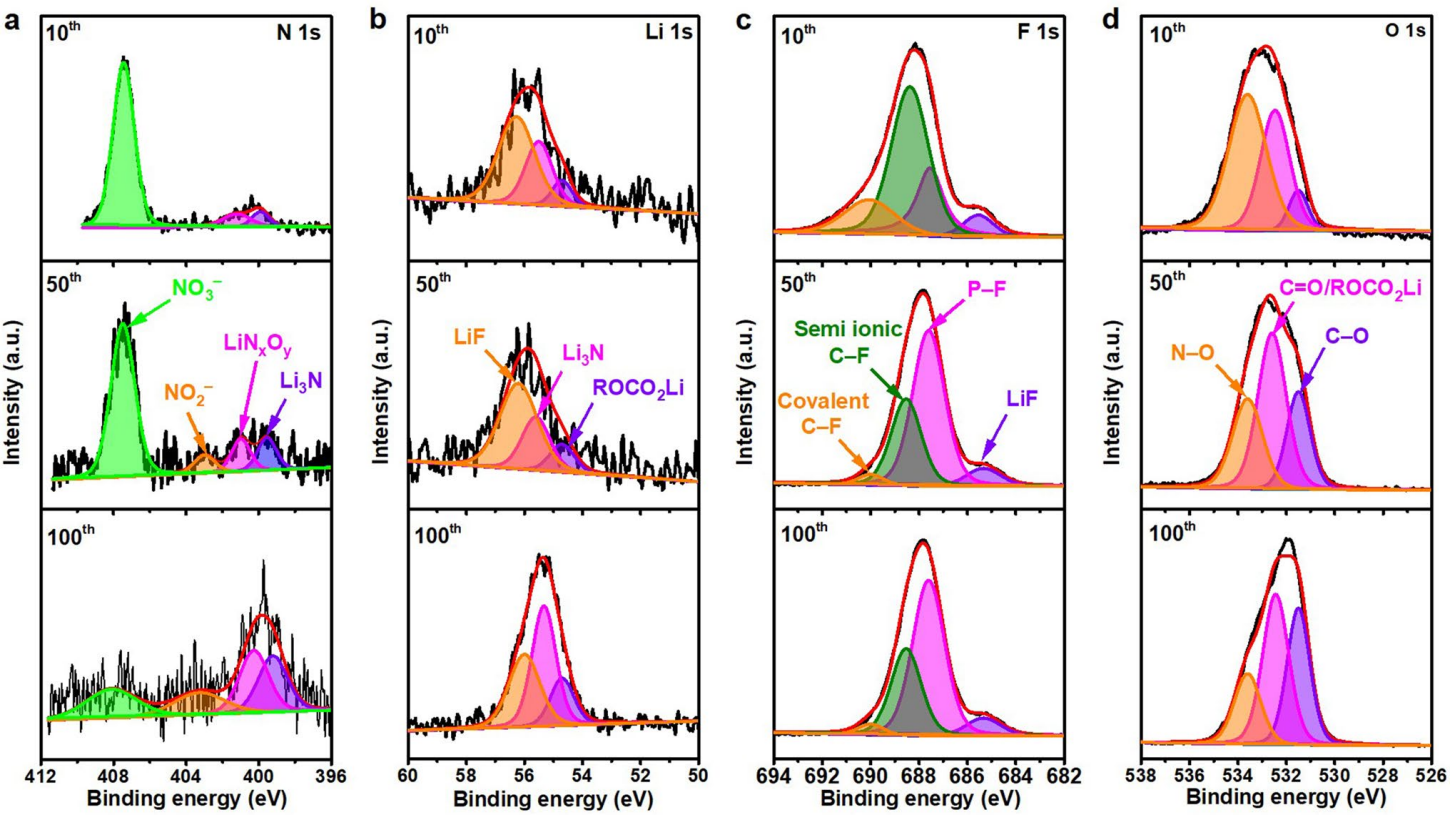
XPS characterization of the SEI films for the PHL-Cu electrodes after different cycles in Li||Cu cells: **a** N 1*s*, **b** Li 1*s* **c** F 1*s* and **d** O 1*s*

XPS was applied to detect the element component of the Cu electrode after various cycles of Li plating/stripping behavior. The main elements of the surface for the bare Cu electrode are Li, C, O, P and F, while the elements of the surface with PHL-Cu electrode are Li, C, O, N, P and F (Fig. S20). In the N 1*s* spectra (Fig. 5a), the peaks at 399.6, 400.8, 403.2 and 407.5 eV are assigned to Li_3_N, LiN_*x*_O_*y*_, NO_2_^−^ and NO_3_^−^, respectively, which originated from the degradation of LiNO_3_ and agree with the previous reports [28, 42, 53]. With further cycling, the Li_3_N, LiN_*x*_O_*y*_, NO_2_^−^ increased, while the NO_3_^−^ decreased, demonstrating the polymer framework can serve as a reservoir for gradually releasing LiNO_3_ into the ester electrolyte. In the Li 1*s* spectra (Fig. 5b), three pronounced peaks at 54.9, 55.5 and 56.0 eV of the PHL-Cu electrode could be related to the ROCO_2_Li, Li−N and LiF, respectively [28]. The intensity of Li−N increased with deeply cycling, further signifying the uninterrupted generation of Li_3_N and LiN_*x*_O_*y*_. The peaks appeared at 54.9, 55.2 and 56.0 eV in Li 1*s* spectrum (Fig. S21a) for the bare Cu electrode of could ascribed to the ROCO_2_Li, Li_2_CO_3_ and LiF, respectively. As for the F 1*s* spectra (Fig. 5c) of the PHL-Cu electrode, four visible peaks located at 685.3, 687.5, 688.3 and 690.2 eV could be related to the LiF, P−F, semi-ionic C−F and covalent C−F bonds, respectively [54]. For the bare Cu electrode, only two binding energies at 685.3 and 687.5 eV correspond to the LiF and P−F bond can be observed in the F 1*s* spectrum (Fig. S21b). For the O 1*s* spectra, the PHL-Cu electrode (Fig. 5d) presented an additional N−O peak at 533.6 eV compared to that of the bare Cu electrode (Fig. S21c), further support the generation of nitrogen-containing species [55]. Specially, the intensity of Li_2_CO_3_ for PHL-Cu electrode was lower than that of the bare Cu electrode, indicating the side reactions with electrolyte were greatly reduced and can be also verified in the C 1*s* spectra (Fig. S22). The continuously nitrogen-containing species, generated by the reduction of LiNO_3_, are capable of improving the stability and ionic conductivity of the SEI layer, which conduce to suppress Li dendrites and expedite Li-ion transport, thus achieving uniform Li deposition.

### Electrochemical Performance of PHL-Cu@Li anode in Practical LMB

**Fig. 6:**
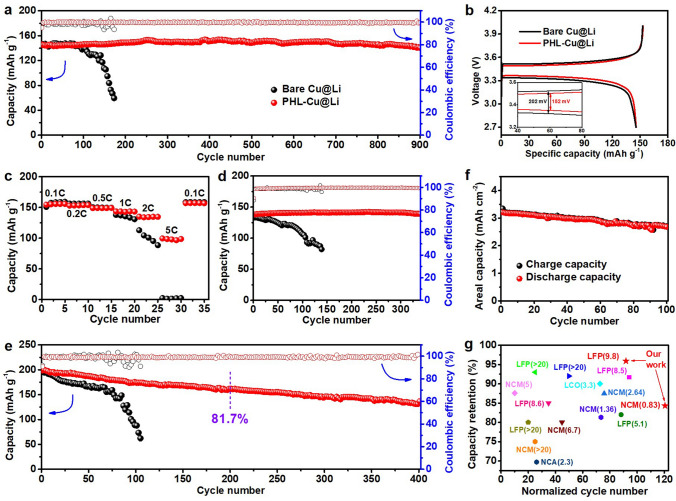
**a** Cycling stability and **b** initial charge/discharge curve of bare Cu@Li||LFP and PHL-Cu@Li||LFP full cells at 0.5C; **c** rate capability of bare Cu@Li||LFP and PHL-Cu@Li||LFP full cells; **d** Cycling stability of bare Cu@Li||LFP and PHL-Cu@Li||LFP full cells at 1C; **e** Cycling stability of bare Cu@Li||NCM and PHL-Cu@Li||NCM full cells at 0.5C; **f** Cycling stability of PHL-Cu@Li||NCM full cell under a ultralow N/P ratio of 0.83 at 0.5C; **g** Comparison of capacity retention and the corresponding cycle life of the PHL-Cu@Li in practical LMBs (The normalized cycle number refers to the ratio of total cycle number to the N/P value)

To survey the practical value of PHL in LMBs, the Li anodes coupled with commercial cathode (LFP or NCM) were employed to assemble full cells. The bare Cu@Li and PHL-Cu@Li anodes were obtained by respectively depositing a certain amount of Li on bare Cu and PHL-Cu electrodes at 0.5 mA cm^−2^. As can be seen in Fig. 6a, the PHL-Cu@Li||LFP cell exhibited long cycle life with admirable capacity retention of 95.9% and high average CE of 99.7% over 900 cycles at 0.5C (1C = 170 mAh g^−1^, N/P = 9.8). By contrast, the bare Cu@Li||LFP cell presented inferior cycle performance less than 200 cycles (capacity retention is only ~ 40.1%) and undulant CE. Figure 6b depicts the initial charge/discharge profile of Li||LFP full cell in a voltage range of 2.7−4.0 V. The PHL modified cell delivered a similar discharge capacity (145.7 vs. 145.6 mAh g^−1^) but lower polarization compared to the bare Cu@Li||LFP cell, indicating a faster Li-ion transport realized by the protective layer. For the rate capability test (Figs. 6c and S23), the discharge capacity of the PHL-Cu@Li anode is higher than that of the bare Cu@Li anode at high C rate. When the charge/discharge rate rise to 1C and 2C, the PHL-Cu@Li||LFP cell still displayed excellent cycle stability over 340 and 150 cycles (Figs. 6d and S24). A relatively small charge transfer impedance for the PHL-Cu@Li||LFP cell after rate capability tests could be obtained from the EIS results (Fig. S25), indicating the formation of a more stable interface, which can be also verified by the SEM results (Fig. S26). To further confirm the availability of PHL layer for high-energy–density NCM cathode, the Li||NCM full cell (the N/P is 5.8) were assembled and cycled with a voltage range of 2.8–4.3 V. As depicted in Figs. 6b and S27, the cell with PHL-Cu@Li anode showed lower overpotential, further proving faster ion transport. The PHL-Cu@Li||NCM cell exhibited an initial capacity of 198.9 mAh g^−1^ at 1C (1C = 200 mAh g^−1^) with a capacity retention of 68.5% after 400 cycles (81.7% for 200 cycles) and an average CE of 99.1%. By contrast, the Cu@Li||NCM battery delivered lower specific capacity and worse CE within 110 cycles. Impressively, when the limited PHL-Cu@Li anode (3 mAh cm^−2^) were assembled with high mass loading NCM (18 mg cm^−2^) cathode at lower-level N/P of 0.83, the full cells maintained over 100 cycles with considerable capacity retention of 84.3% at 0.5C (Fig. 6f). The cycle stability and capacity retention of the full cells with PHL-Cu@Li anode outperform most of the reported results, as summarized in Fig. 6g and Table S3. The remarkably prolonged cycle life of Li||LFP and Li||NCM full cells could be ascribed to the PHL layer, which acts as a reservoir to release LiNO_3_ for stable SEI build. The SEI layer enriched with nitrogen-containing species is capable of improving the stability of electrode/electrolyte interface and expediting the transport of Li ion, resulting in uniform Li deposition and dendrite suppression.

## Conclusions

In summary, LiNO_3_-implanted electroactive β phase PVDF-HFP crystalline polymorph layer was built on Li surface for dendrite suppression. The electroactive polymer chains acquire Li ions on its surface to form Li-ion charged channels, which could serve as a reservoir for continuous release of Li ion to recompense the ionic flux of electrolytes. Additionally, the ionic conductive nitrogen-containing species derived from LiNO_3_ enhance as well as the stretched molecular channels could homogenize the Li-ion flux and promote the ion transport, greatly restraining the dendrite growth and achieving uniform Li deposition. Consequently, a higher CE of 97.0% over 250 cycles in ester electrolyte was obtained in a Li||Cu cell, and stable Li plating/stripping behaviors under large current density with high Li utilization were also achieved in symmetric cell. Long life LMBs were achieved by the PHL-Cu@Li anodes and LFP cathodes, which could achieve 95.9% capacity retention after 900 cycles, far exceeding that of the bare Li anode. And the PHL-Cu@Li||NCM full cell also exhibited high discharge capacity and excellent cycling stability even under more realistic condition of low N/P of 0.83. We believe this simple strategy will broaden the application of LiNO_3_ in ester-based electrolyte for high-energy–density LMBs and other metal batteries.

## Supplementary Information

Below is the link to the electronic supplementary material.

Supplementary file 1.

Supplementary file 2.

Supplementary file 3.
