# Supplementary material for: Lithium-Ion Charged Polymer Channels Flattening Lithium Metal Anode
Source: Nanomicro Lett. 2024 Jan 8;16:78. doi: 10.1007/s40820-023-01300-5 (PMC10774241; doi:10.1007/s40820-023-01300-5)
Supplement: Supplementary file 1 [file 40820_2023_1300_MOESM1_ESM.pdf]

Supporting Information for

Lithium-Ion Charged Polymer Channels Flattening Lithium Metal Anode

Haofan Duan[1,#], Yu You[1,#], Gang Wang[1,*], Xiangze Ou[1], Jin Wen[1], Qiao Huang[1], Pengbo Lyu[1], Yaru Liang[1,*], Qingyu Li[2], Jianyu Huang[1], Yun-Xiao Wang[3], Hua-Kun Liu[3,4], Shi Xue Dou[3,4], Wei-Hong Lai[3,*]

[1]Hunan Provincial Key laboratory of Thin Film Materials and Devices, School of Material Sciences and Engineering, Xiangtan University, Xiangtan 411105, P. R. China

[2]Guangxi Key Laboratory of Low Carbon Energy Materials, School of Chemical and Pharmaceutical Science, Guangxi Normal University, Guilin 541004, P. R. China

[3]Institute for Superconducting & Electronic Materials, Australian Institute of Innovative Materials, University of Wollongong, Innovation Campus, Squires Way, North Wollongong, NSW 2500, Australia

[4]Institute of Energy Materials Science, University of Shanghai for Science and Technology, Shanghai 200093, P. R. China

[#]Haofan Duan and Yu You contributed equally to this work.

*Corresponding authors. E-mail: esgwang@xtu.edu.cn (Gang Wang); yaruliang@xtu.edu.cn (Yaru Liang); weihongl@uow.edu.au (Wei-Hong Lai)

Supplementary Figures and Tables

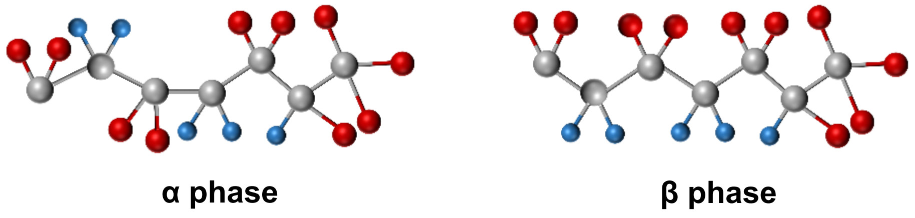

Fig. S1 Diagrams of the chain conformation for α and β crystalline phases of PVDF-HFP

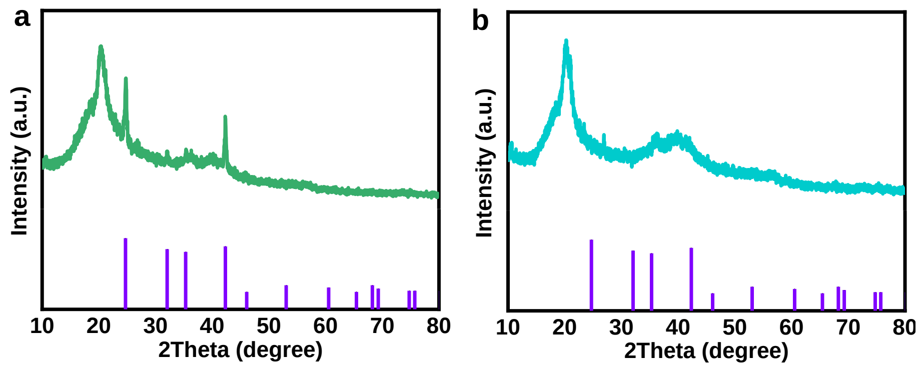

Fig. S2 XRD patterns of PHL with mass ratios of **a** 1:1 and **b** 1:5

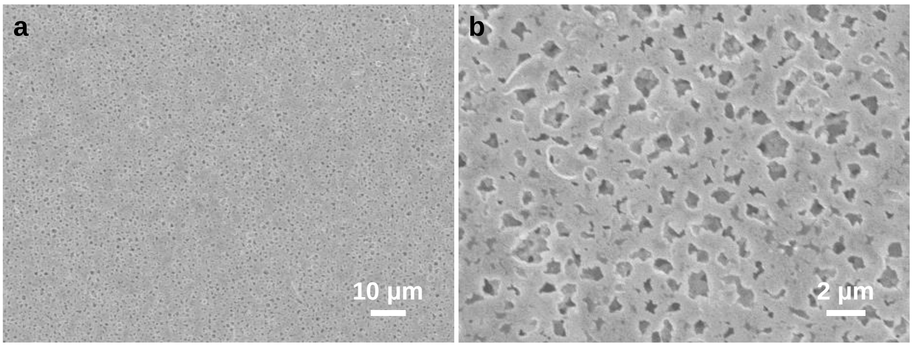

Fig. S3 SEM images of the pure PVDF-HFP (PH) at different magnifications

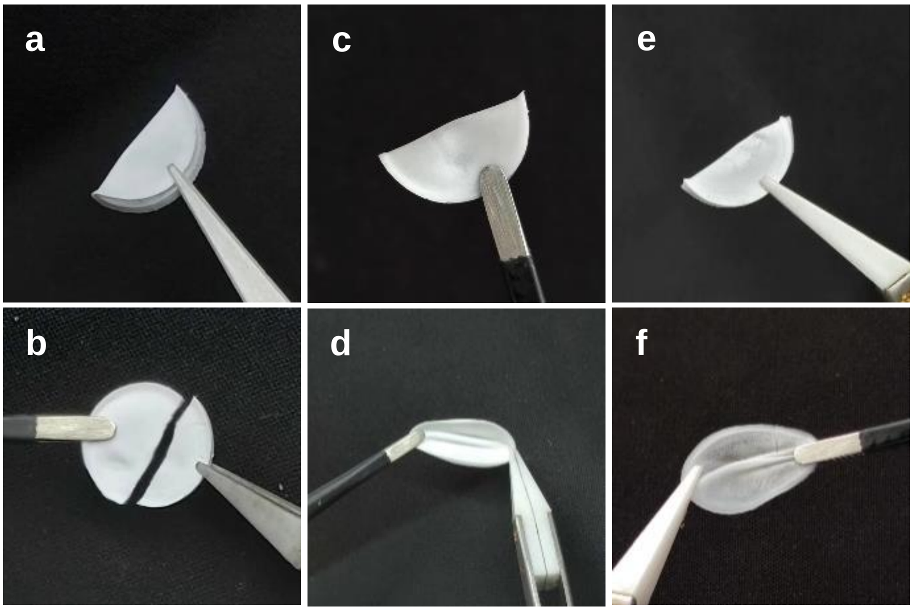

Fig. S4 Optical photographs of PHL films after folding and stretching with different mass ratios of **a b** 1:1, **c d** 1:3 and **e f** 1:5

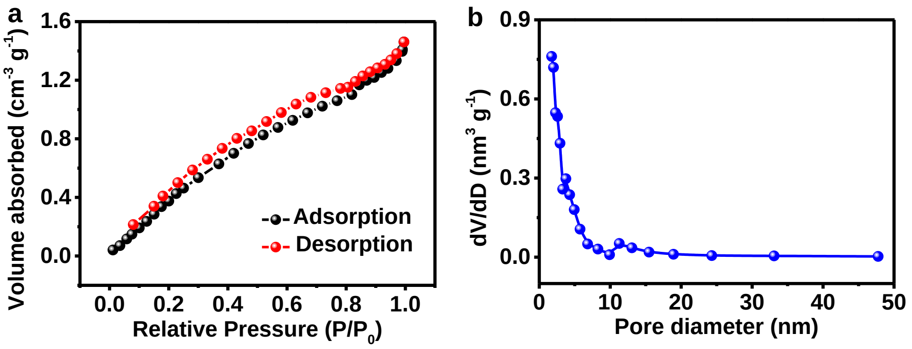

Fig. S5 a N_2 adsorption/desorption isotherm and **b** corresponding pore size distribution of PHL

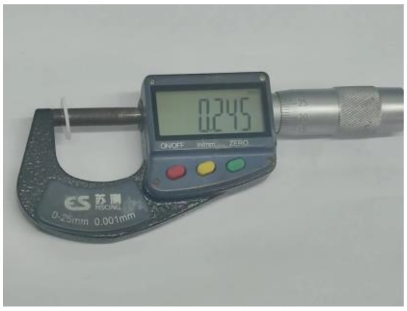

Fig. S6 The thickness of the PHL composite layer with various mass ratio of 1:3

Table S1 Ionic conductivity of PVDF-HFP/$LiNO_3$ composites film with various mass ratios

Mass ratio	Area (cm^2)	Thickness (mm)	Ohmic resistance (Ω)	Ionic conductivity (S cm^{-1})
1:1	2.01	0.250	298	4.17×10^{-5}
3:1	2.01	0.245	36	3.39×10^{-4}
5:1	2.01	0.248	159	7.76×10^{-5}

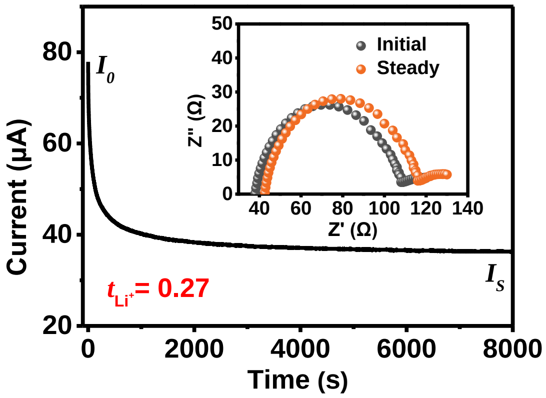

Fig. S7 Chronoamperomtric curves and corresponding EIS before/after polarization of bare Li

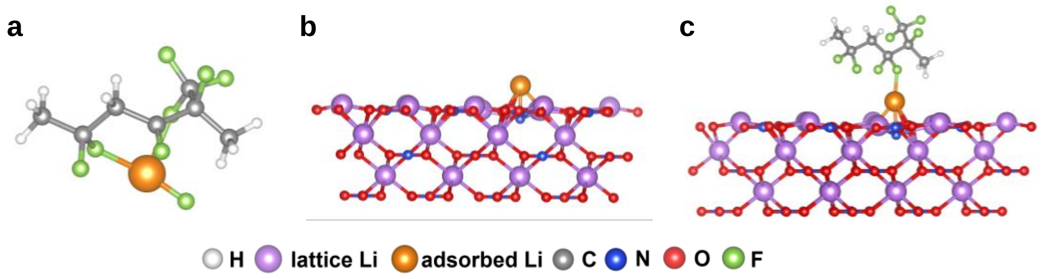

Fig. S8 Optimized geometrical structures of Li atoms adsorbed on **a** PH, **b** $LiNO_3$, and **c** PHL

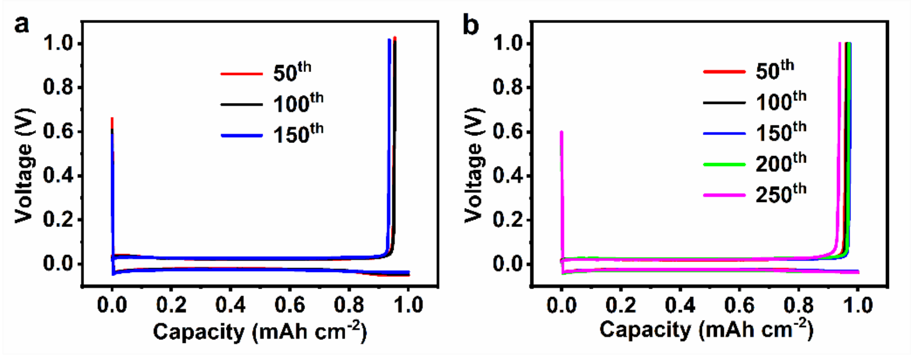

Fig. S9 Charge/discharge curves of Li||Cu cells using **a** bare Cu and **b** PHL-Cu at 0.5 mA cm^{-2}

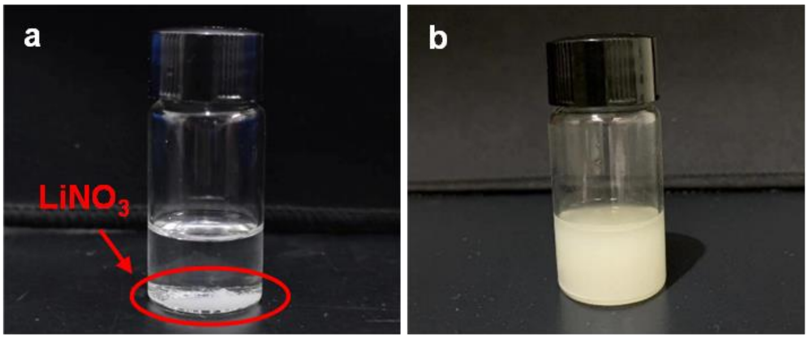

Fig. S10 Optical photographs of $LiNO_3$ dissolution test in the ester-based electrolyte: **a** before and **b** after magnetic stirring for 12 h

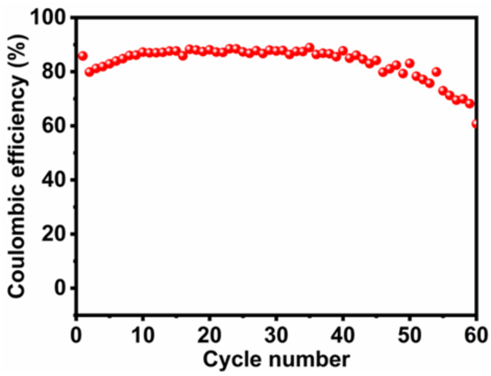

Fig. S11 CE of Li||Cu cell using $LiNO_3$ as electrolyte additive at a current density of 1 mA cm^{-2} under a fixed capacity of 1 mAh cm^{-2}

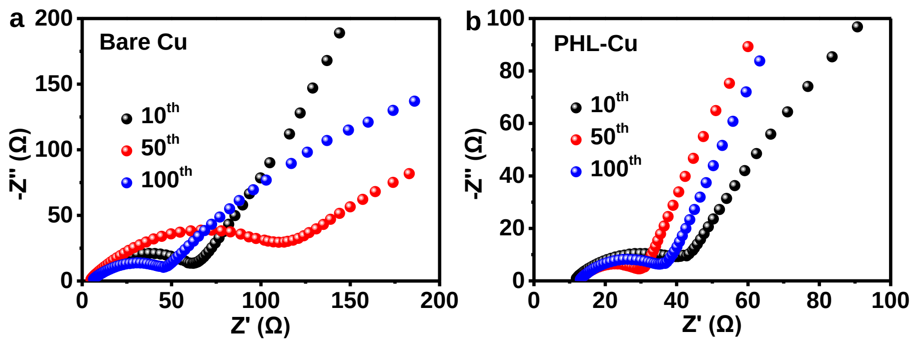

Fig. S12 EIS plots of Li||Cu cells using **a** bare Cu and **b** PHL-Cu after different cycles at a current density of 1 mA cm^{-2} under 1 mAh cm^{-2}

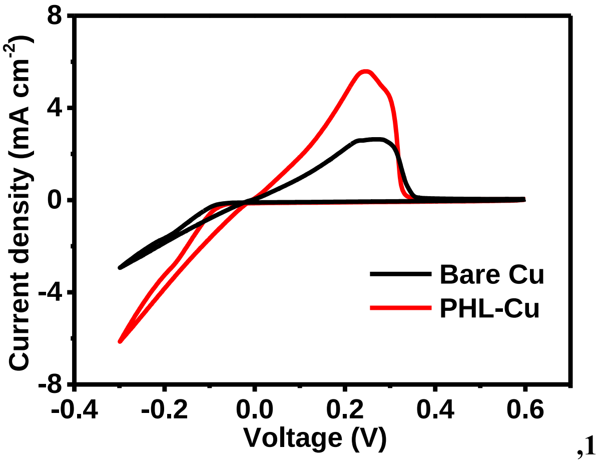

Fig. S13 CV of Li||Cu cells using bare Cu and PHL-Cu recorded at a scan rate of 5 mV s^{-1} in a voltage range of -0.3–0.6 V

As shown in Fig. S13, the current response of the PHL-Cu electrode is greatly high than that of the bare Cu electrode during Li plating/stripping process, indicating the fast Li^+ transport and reversible reaction kinetics of the Li||PHL-Cu cell.

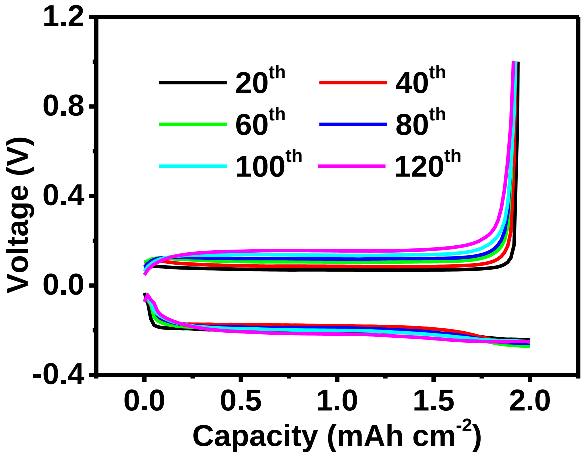

Fig. S14 Charge/discharge curves of Li||Cu cells using PHL-Cu in particular cycles at 2 mA cm^{-2}

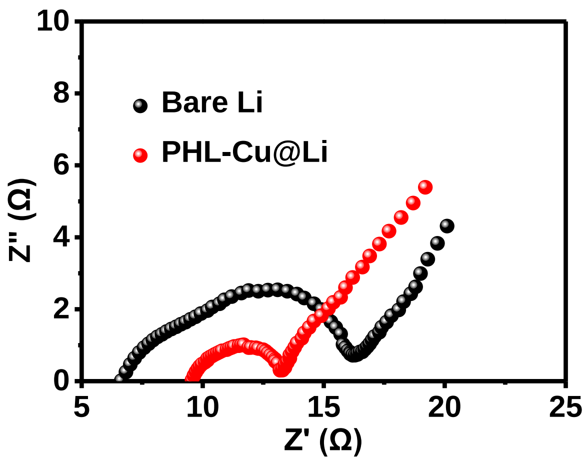

Fig. S15 EIS of bare Li and PHL-Cu@Li anodes after 50 cycles of Li plating/stripping at 1 mA cm^{-2} to 1 mAh cm^{-2}

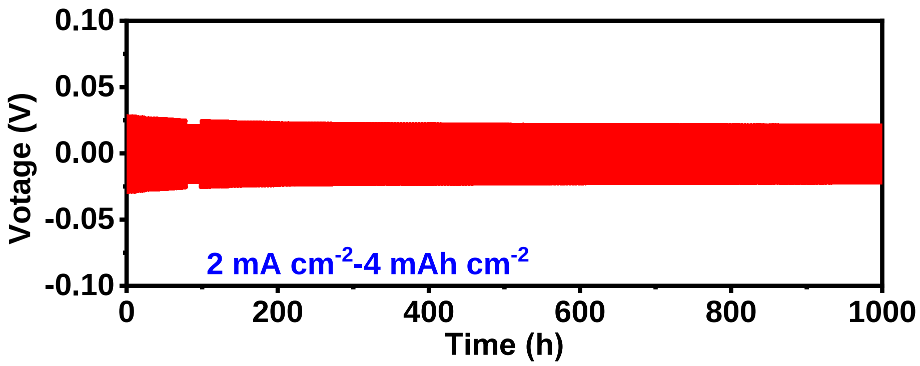

Fig. S16 Voltage profiles of Li||Li symmetric cells with PHL-Cu@Li at 2 mA cm^{-2} under high capacity of 4 mAh cm^{-2}

Table S2 Li plating/stripping behavior of the PHL-Cu@Li anode at various current density-capacity conditions with previously reports on the $LiNO_3$ dissolution in ester-based electrolyte

Strategy	Current density (mA cm^{-2})	Capacity (mAh cm^{-2})	Cycle time (h)	Electrolyte	Ref.
FEC-SL/$LiNO_3$	3	1	200	1 M $LiPF_6$ in EC/DMC	[S1]
MgAl-LDHs	0.5	1	500	1 M $LiPF_6$ in EC/DMC/DEC+5%FEC	[S2]
Pyridine	1	1	300	1 M $LiPF_6$ in EC/EMC/DMC	[S3]
Tetraglyme	1	1	800	1 M $LiPF_6$ in EC/DMC	[S4]
EB-COF:NO_3	5	10	300	1 M $LiPF_6$ in EC/EMC	[S5]
IL–NO_3^-	1	1	600	1 M $LiPF_6$ in EC/DMC/EMC	[S6]
TEAN	1	1	1100	1 M $LiPF_6$ in EC/DEC+10% FEC	[S7]

LNO-PVC	2	1	300	1 M $LiPF_6$ in EC/DMC +5%FEC	[S8]
LN-CPL	0.5	0.5	300	1.15 M $LiPF_6$ in EC/EMC	[S9]
LNO@MOF	1	1	1000	1 M $LiPF_6$ in EC/DEC	[S10]
PHL	1	1	3000	1 M $LiPF_6$ in EC/DMC/EMC+5%FEC	Our work
	3	1	2000		
	5	1	1000		
	3	3	2000		
	2	4	800		

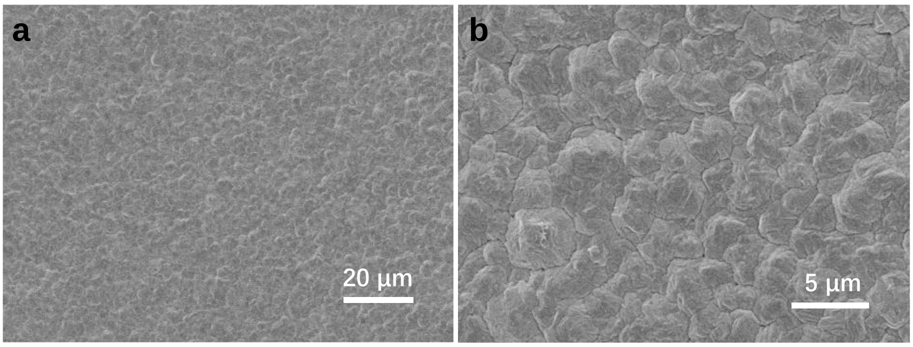

Fig. S17 SEM image of bare Cu electrode under different magnification

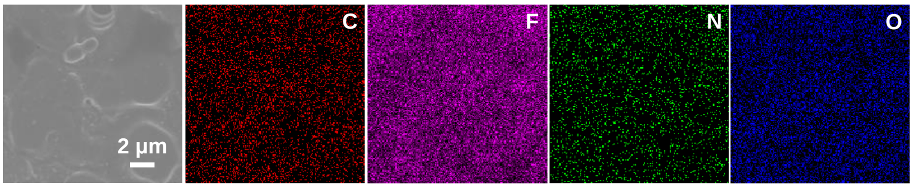

Fig. S18 SEM images of PHL-Cu electrode and corresponding EDS mappings in Li||Cu cells after 50 cycles

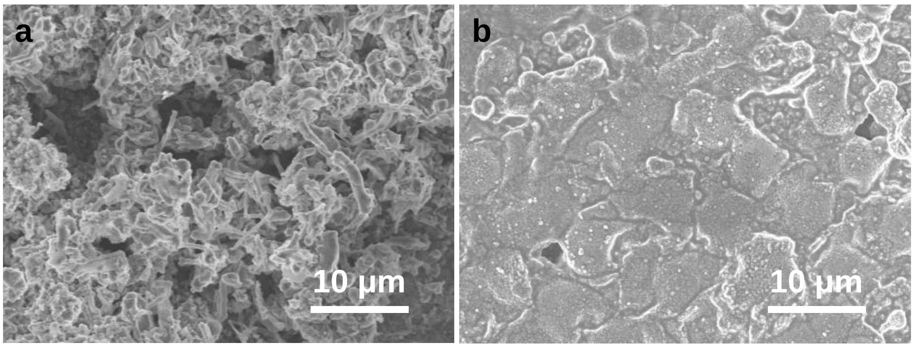

Fig. S19 SEM images of Li||Cu cells using **a** bare Cu and **b** PHL-Cu after 100 cycles

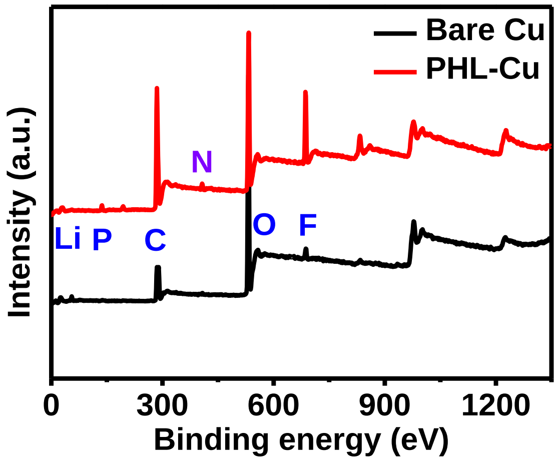

Fig. S20 Survey XPS spectra of bare Cu and PHL-Cu electrodes after 10 cycles in Li||Cu cells

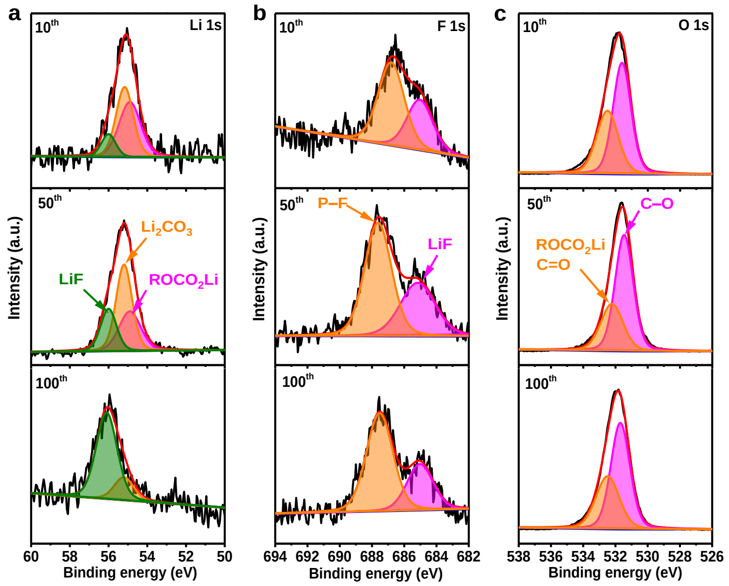

Fig. S21 XPS characterization of the SEI films for the bare Cu electrodes after different cycles in Li||Cu cells: **a** Li 1s, **b** F 1s and **c** O 1s

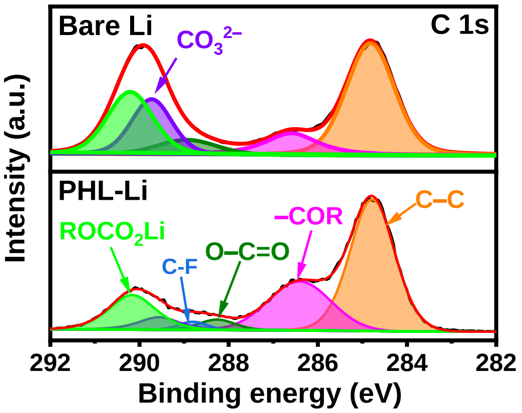

Fig. S22 Comparison of high-resolution XPS spectra of C 1 s for bare Cu and PHL-Cu electrodes after 100 cycles

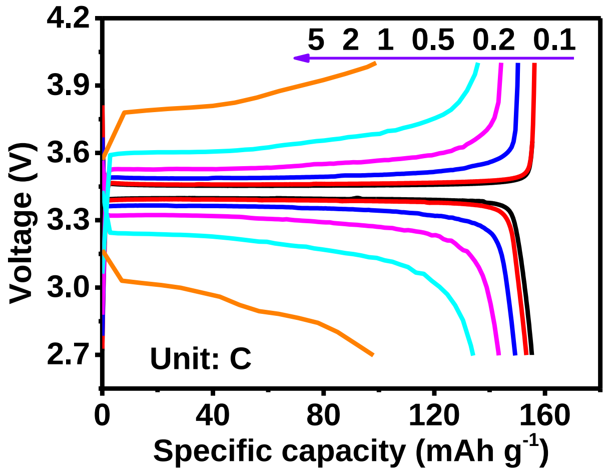

Fig. S23 The charge/discharge profiles of the PHL-Cu@Li||LFP cell at different C-rate

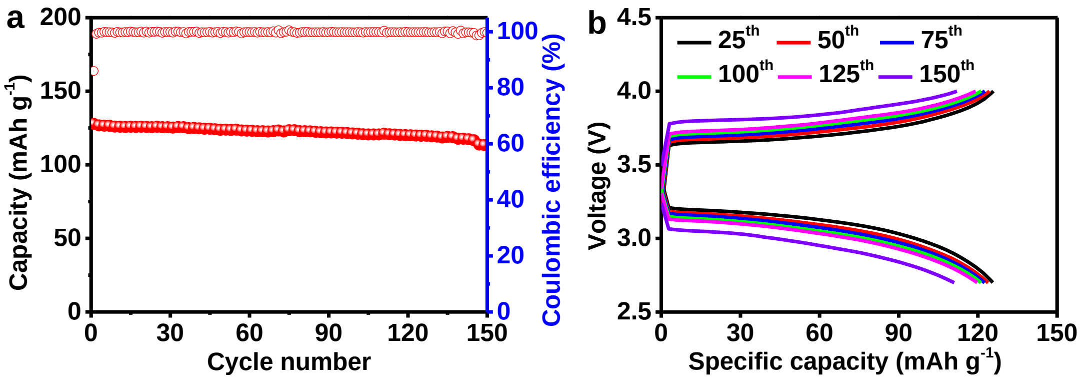

Fig. S24 a Cycle performance and **b** corresponding charge/discharge profiles at particular cycles of the PHL-Cu@Li||LFP cell at 2C

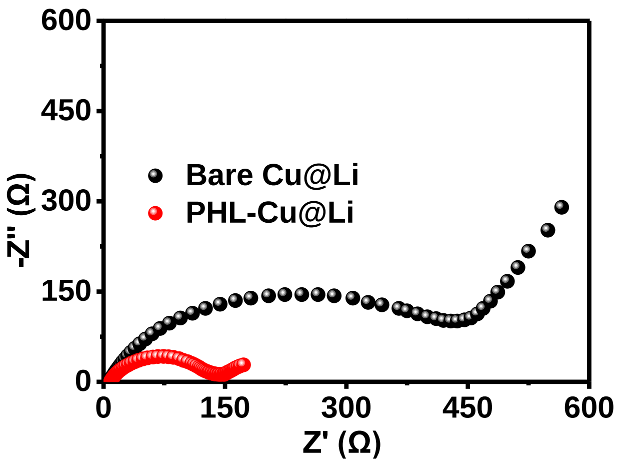

Fig. S25 EIS of the Li||LFP cells with bare Cu@Li and PHL-Cu@Li after rate capability test

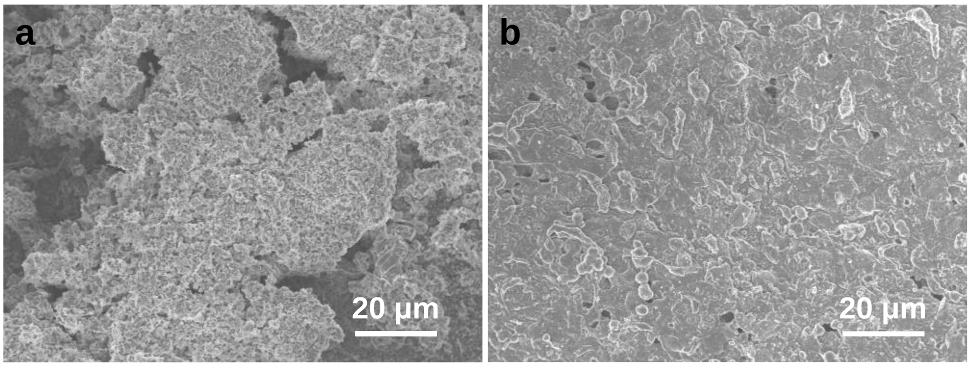

Fig. S26 SEM images of the **a** bare Cu@Li and **b** PHL-Cu@Li electrodes after 100 cycles in Li||LFP full cell

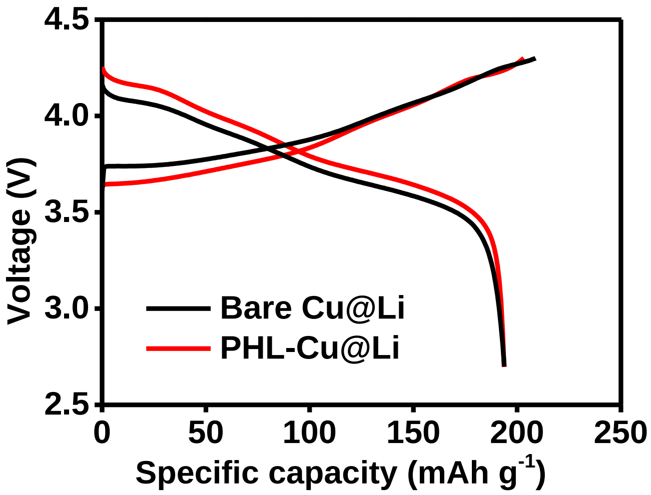

Fig. S27 Initial charge/discharge profiles of the PHL-Cu@Li||NCM cell at 0.5C

Table S3 Summary of cycling performance of LMBs with low N/P ratios and Li utilization efficiency in literature

Cathode	N/P ratio	Cycle number	Capacity retention	Rate density	Li utilization efficiency	Refs.
LFP	8.5	800	91.7%	2 C	11.76%	[S11]
LFP	5.1	450	82%	0.5 C	19.61%	[S12]
NCM811	6.7	300	80%	C/3	14.93%	[S13]
LCO	3.3	240	90%	0.2 C/0.5 C	30.30%	[S10]
LFP	＞20	500	93%	2 C	＜5%	[S14]
LFP	＞20	1000	92%	1 C	＜5%	[S15]
NCM811	＞20	500	75%	0.5 C	＜5%	
LFP	＞20	400	80%	0.5 C	＜5%	[S16]
NCA	2.3	60	69.7%	0.3 C	43.48%	[S17]
NCM811	2.64	200	87.5%	0.3 C	37.88%	[S18]
LFP	8.6	300	85%	1 C	11.63%	[S19]
LNCM	5	50	87.6%	1 C	20%	[S20]

NCM811	1.36	100	81.3%	1 C	73.53%	[S21]
LFP	9.8	900	97%	0.5 C	10.20%	Our
NCM87	0.83	100	85%	0.5 C	120.48%	work

Supplementary References

[S1] N. Piao, S. Liu, B. Zhang, X. Ji, X. Fan et al., Lithium metal batteries enabled by synergetic additives in commercial carbonate electrolytes. ACS Energy Lett. **6**, 1839–1848 (2021). https://doi.org/10.1021/acsenergylett.1c00365

[S2] F. Wang, Z. Wen, Z. Zheng, W. Fang, L. Chen et al., Memory effect of MgAl layered double hydroxides promotes $LiNO_3$ dissolution for stable lithium metal anode. Adv. Energy Mater. **13**, 2203830 (2023). https://doi.org/10.1002/aenm.202203830

[S3] D. Liu, X. Xiong, Q. Liang, X. Wu, H. Fu, An inorganic-rich SEI induced by $LiNO_3$ additive for a stable lithium metal anode in carbonate electrolyte. Chem. Commun. **57**, 9232–9235 (2021). https://doi.org/10.1039/D1CC03676A

[S4] D. Xiao, Q. Li, D. Luo, G. Li, H. Liu et al., Regulating the Li^+-solvation structure of ester electrolyte for high-energy-density lithium metal batteries. Small **16**, e2004688 (2020). https://doi.org/10.1002/smll.202004688

[S5] Y. Wen, J. Ding, Y. Yang, X. Lan, J. Liu et al., Introducing NO_3^- into carbonate-based electrolytes via covalent organic framework to incubate stable interface for Li-metal batteries. Adv. Funct. Mater. **32**, 2109377 (2022). https://doi.org/10.1002/adfm.202109377

[S6] X. Ma, J. Yu, X. Zou, Y. Hu, M. Yang et al., Single additive to regulate lithium-ion solvation structure in carbonate electrolytes for high-performance lithium-metal batteries. Cell Rep. Phys. Sci. **4**, 101379 (2023). https://doi.org/10.1016/j.xcrp.2023.101379

[S7] Z. Guo, X. Song, Q. Zhang, N. Zhan, Z. Hou et al., Cationic size effect promoting dissolution of nitrate anion in ester electrolyte for lithium–metal batteries. ACS Energy Lett. **7**, 569–576 (2022). https://doi.org/10.1021/acsenergylett.1c02495

[S8] H. Yang, Q. Liu, Y. Wang, Z. Ma, P. Tang et al., An interlayer containing dissociated $LiNO_3$ with fast release speed for stable lithium metal batteries with 400 wh kg^{-1} energy density. Small **18**, e2202349 (2022). https://doi.org/10.1002/smll.202202349

[S9] J.-T. Kim, I. Phiri, S.-Y. Ryou, Incorporation of embedded protective layers to circumvent the low $LiNO_3$ solubility problem and enhance Li metal anode cycling performance. ACS Appl. Energy Mater. **6**, 2311–2319 (2023). https://doi.org/10.1021/acsaem.2c03511

[S10] Q. Liu, Y. Xu, J. Wang, B. Zhao, Z. Li et al., Sustained-release nanocapsules enable long-lasting stabilization of Li anode for practical Li-metal batteries. Nanomicro Lett. **12**, 176 (2020). https://doi.org/10.1007/s40820-020-00514-1

[S11] D. Huang, C. Zeng, M. Liu, X. Chen, Y. Li et al., Introducing KI as a functional electrolyte additive to stabilize Li metal anode. Chem. Eng. J. **454**, 140395 (2023). https://doi.org/10.1016/j.cej.2022.140395

[S12] Z. Jiang, C. Li, J. Mo, H. Yang, H.-W. Li et al., A cation-anion synergetic additive achieving long-term stability of lithium metal anode. Chem. Eng. J. **451**, 138580 (2023). https://doi.org/10.1016/j.cej.2022.138580

[S13] X. Cao, X. Ren, L. Zou, M.H. Engelhard, W. Huang et al., Monolithic solid–electrolyte interphases formed in fluorinated orthoformate-based electrolytes minimize Li depletion and pulverization. Nat. Energy **4**, 796–805 (2019). https://doi.org/10.1038/s41560-019-0464-5

[S14] Z. Hu, S. Zhang, S. Dong, W. Li, H. Li et al., Poly(ethyl α-cyanoacrylate)-based artificial solid electrolyte interphase layer for enhanced interface stability of Li metal anodes. Chem. Mater. **29**, 4682–4689 (2017). https://doi.org/10.1021/acs.chemmater.7b00091

[S15] Z. Cheng, Y. Chen, L. Shi, M. Wu, Z. Wen, Long-lifespan lithium metal batteries enabled by a hybrid artificial solid electrolyte interface layer. ACS Appl. Mater. Interfaces **15**, 10585–10592 (2023). https://doi.org/10.1021/acsami.2c18224

[S16] C. Yan, Y.-X. Yao, X. Chen, X.-B. Cheng, X.-Q. Zhang et al., Lithium nitrate solvation chemistry in carbonate electrolyte sustains high-voltage lithium metal batteries. Angew. Chem. Int. Ed Engl. **57**, 14055–14059 (2018). https://doi.org/10.1002/anie.201807034

[S17] Y. Liu, X. Qin, D. Zhou, H. Xia, S. Zhang et al., A biscuit-like separator enabling high performance lithium batteries by continuous and protected releasing of NO3–in carbonate electrolyte. Energy Storage Mater. **24**, 229–236 (2020). https://doi.org/10.1016/j.ensm.2019.08.016

[S18] W. Zhang, Q. Wu, J. Huang, L. Fan, Z. Shen et al., Colossal granular lithium deposits enabled by the grain-coarsening effect for high-efficiency lithium metal full batteries. Adv. Mater. **32**, e2001740 (2020). https://doi.org/10.1002/adma.202001740

[S19] Y. Cui, S. Liu, D. Wang, X. Wang, X. Xia et al., A facile way to construct stable and ionic conductive lithium sulfide nanoparticles composed solid electrolyte interphase on Li metal anode. Adv. Funct. Mater. **31**, 2006380 (2021). https://doi.org/10.1002/adfm.202006380

[S20] X. Yan, L. Lin, X. Han, Z. Qiao, Q. Xie et al., Li dendrites inhibition realized by lithiophilic and ion/electron conductive 3D skeleton for Li metal anodes. Chem. Eng. J. **421**, 127872 (2021). https://doi.org/10.1016/j.cej.2020.127872

[S21] S. Cui, P. Zhai, W. Yang, Y. Wei, J. Xiao et al., Large-scale modification of commercial copper foil with lithiophilic metal layer for Li metal battery. Small **16**, e1905620 (2020). https://doi.org/10.1002/smll.201905620
